# Presepsin (Soluble CD14 Subtype) as an Early Marker of Neonatal Sepsis and Septic Shock: A Prospective Diagnostic Trial

**DOI:** 10.3390/antibiotics10050580

**Published:** 2021-05-14

**Authors:** Carlo Pietrasanta, Andrea Ronchi, Claudia Vener, Chiara Poggi, Claudia Ballerini, Lea Testa, Rosaria Maria Colombo, Elena Spada, Carlo Dani, Fabio Mosca, Lorenza Pugni

**Affiliations:** 1 Fondazione IRCCS Ca’ Granda Ospedale Maggiore Policlinico, NICU, 20122 Milan, Italy; andrea.ronchi@policlinico.mi.it (A.R.); claudiaballerini28@gmail.com (C.B.); lea.tst95@gmail.com (L.T.); elenaspada.bios@gmail.com (E.S.); fabio.mosca@unimi.it (F.M.); lorenza.pugni@mangiagalli.it (L.P.); 2 Department of Clinical Sciences and Community Health, University of Milan, 20122 Milan, Italy; 3 Department of Oncology and Hemato-Oncology, University of Milan, 20122 Milan, Italy; claudia.vener@unimi.it; 4 Division of Neonatology and Neonatal Intensive Care, Department of Mother and Child Care, Careggi University Hospital, 50141 Florence, Italy; poggich@gmail.com (C.P.); carlo.dani@unifi.it (C.D.); 5 Clinical Laboratory, Fondazione IRCCS Ca’ Granda Ospedale Maggiore Policlinico, 20122 Milan, Italy; rosaria.colombo@policlinico.mi.it; 6 Department of Neurosciences, Psychology, Drug Research, and Child Health, University of Florence, 50139 Florence, Italy

**Keywords:** newborn, neonatal sepsis, septic shock, presepsin, biomarkers, inflammation

## Abstract

In the context of suspected neonatal sepsis, early diagnosis and stratification of patients according to clinical severity is not yet effectively achieved. In this diagnostic trial, we aimed to assess the accuracy of presepsin (PSEP) for the diagnosis and early stratification of supposedly septic neonates. PSEP, C-reactive protein (CRP), and procalcitonin (PCT) were assessed at the onset of sepsis suspicion (T0), every 12–24 h for the first 48 h (T1–T4), and at the end of antibiotic therapy (T5). Enrolled neonates were stratified into three groups (infection, sepsis, septic shock) according to Wynn and Wong’s definitions. Sensitivity, specificity, and area under the ROC curve (AUC) according to the severity of clinical conditions were assessed. We enrolled 58 neonates with infection, 77 with sepsis, and 24 with septic shock. PSEP levels were higher in neonates with septic shock (median 1557.5 pg/mL) and sepsis (median 1361 pg/mL) compared to those with infection (median 977.5 pg/mL) at T0 (*p* < 0.01). Neither CRP nor PCT could distinguish the three groups at T0. PSEP’s AUC was 0.90 (95% CI: 0.854–0.943) for sepsis and 0.94 (95% CI: 0.885–0.988) for septic shock. Maximum Youden index was 1013 pg/mL (84.4% sensitivity, 88% specificity) for sepsis, and 971.5 pg/mL for septic shock (92% sensitivity, 86% specificity). However, differences in PSEP between neonates with positive and negative blood culture were limited. Thus, PSEP was an early biomarker of neonatal sepsis severity, but did not support the early identification of neonates with positive blood culture.

## 1. Introduction

Despite a favorable trend over the last two decades, neonatal sepsis is still a major cause of morbidity, mortality, and antibiotic use among neonates, with an incidence of 1–4 per 1000 live births rising up to 12–17% in very low birth weight infants (VLBWi) [1]. Neonatal septic shock, a severe clinical evolution of sepsis, has a reported incidence of 1–2% in neonatal intensive care units (NICUs) [2,3], with mortality rates up to 70% [4]. Neonates and children with septic shock present hemodynamic differences compared to their adult counterparts, but share with them the rapid derangement towards a compromised clinical condition within a few hours from the onset of symptoms; this frequently occurs independently from the institution of empiric broad spectrum antibiotic therapy [5,6]. The definition of neonatal sepsis is still equivocal [7], and no consensus-based approach akin to Sepsis-3 for adult patients has been developed. At present, blood culture remains the gold standard for the diagnosis of neonatal sepsis, but roughly 48–72 h are needed to obtain a reliable response, and the number of false negative results is not negligible [8]. Furthermore, and independently from blood culture results, a prompt stratification of risk for adverse clinical evolution would be advisable, to allow a timely institution of the more appropriate therapeutic management. In this context, several biomarkers have been repeatedly investigated, such as C-reactive protein (CRP), procalcitonin (PCT), and various interleukins [9]. A fraction of the soluble form of CD14, named soluble CD14 subtype (sCD14-ST) or presepsin (PSEP), has received increasing attention over the past 10 years as a possible early marker of sepsis [10]. In neonates, PSEP seems to ensure a high sensitivity and specificity for the diagnosis of sepsis [11]. Nonetheless, four recent meta-analyses agreed on the weaknesses affecting the majority of published studies investigating PSEP in the context of neonatal sepsis, such as small sample sizes, ambiguous definitions, or absence of stratification for disease severity [11,12,13,14]. We have previously established the reference ranges for PSEP in two large cohorts of healthy term and preterm neonates [15,16]. Here, we aimed to evaluate the diagnostic performance of PSEP for neonatal sepsis, compared to CRP and PCT. In particular, we investigated whether PSEP could support the early identification of neonates with the most unfavorable clinical evolution, towards sepsis or septic shock, and we analyzed if early high values of PSEP were associated with a positive blood culture.

## 2. Results

### 2.1. Clinical Characteristics and Kinetics of Biomarkers

Over the study period, 159 neonates with suspected sepsis were enrolled. Fifty-eight of them were categorized as “infection”, 77 had sepsis, and 24 developed septic shock within 24 h from the onset of symptoms. Baseline characteristics of neonates enrolled are reported in Table 1.

**Figure 1:**
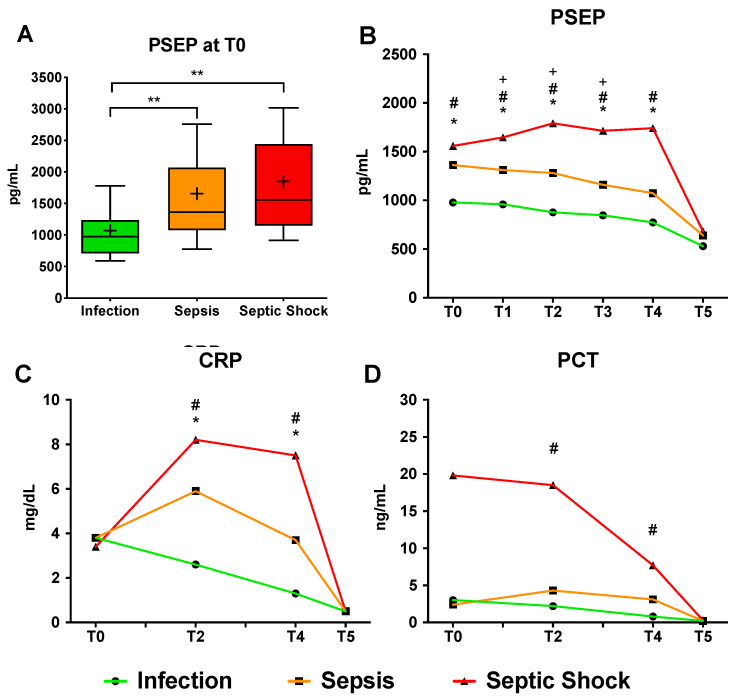
(**A**) Median PSEP levels at T0 (onset of symptoms) in the 3 groups of enrolled neonates. **, *p* < 0.01 after ANOVA with Dunn’s multiple comparisons test. Boxes indicate IQR, whiskers indicate 10° and 90° percentile, crosses indicate means. (**B**–**D**) Serial values (median) of PSEP, CRP and PCT in the 3 groups of neonates. T0: onset of symptoms, T1: 12 h, T2: 24 h, T3: 36 h, T4: 48 h, T5: end of antibiotic therapy. Repeated measures ANOVA with Tukey’s correction for multiple comparisons, *p*-values < 0.01 are marked as follows: *, infection vs. sepsis; #, infection vs. septic shock; +, sepsis vs. septic shock.

PSEP levels at T0 were significantly higher in neonates with sepsis (median 1361 pg/mL, IQR 1082–2065) and septic shock (median 1557.5, IQR 1149.5–2386) compared to those with infection (median 977.5, IQR 709–1239), *p* < 0.001 (Table 1, Figure 1A). 

Conversely, at T0, median blood levels of CRP and PCT were not significantly different between the three groups (Table 1, Figure 1B–D and Supplementary Figure S1) and did not support the early identification of subsequently severely ill patients. The overall correlation between PSEP and other biomarkers at T0 in the three groups of neonates enrolled was weak (Supplementary Figure S2), except for that with PCT in neonates with septic shock (Pearson’s r = 0.44, *p*-value = 0.03). During the first 48 h from the onset of symptoms, PSEP progressively increased in neonates with septic shock, while it remained stable or decreased in neonates with sepsis or infection (Table 2, and Figure 1B).

From T1 (12 h) to T3 (36 h), neonates with ongoing septic shock had a significantly higher PSEP level compared to both the other groups (all *p* < 0.01). Compared to PSEP, CRP levels were higher at T2 in neonates with shock (median 8.2 mg/dL, IQR 4.2–14.9) and sepsis (median 5.9 mg/dL, 2.2–11.3) compared to those with infection (median 2.6 mg/dL, 1.4–5.5), while PCT was higher at T2 and T4 only in neonates with shock compared to those with infection.

### 2.2. Diagnostic Accuracy of Presepsin for Neonatal Sepsis

**Figure 2:**
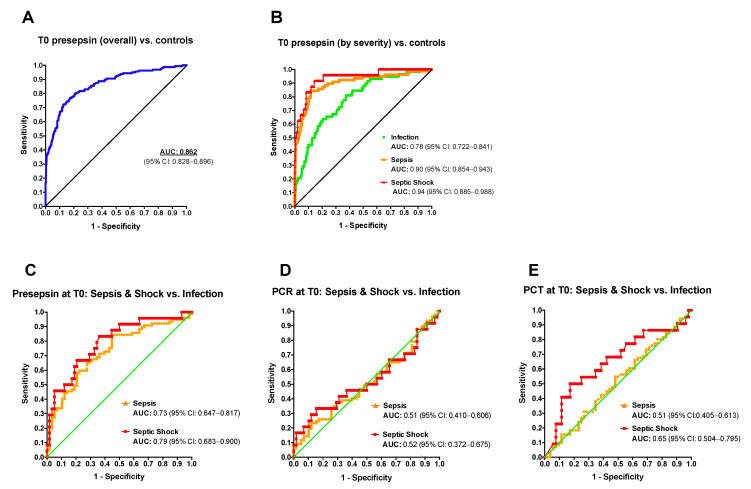
PSEP had a better performance in the early diagnosis of sepsis and septic shock compared to CRP and PCT. (**A**) ROC curves for PSEP at T0 in the overall population and (**B**) in the 3 different groups of neonates (control group = healthy neonates, [16]). ROC curves of PSEP (**C**), CRP (**D**) and PCT (**E**) at T0 in neonates with sepsis (group 2, orange line) or septic shock (group 3, red line) compared to neonates with infection (group 1).

The sensitivity and specificity values of PSEP for sepsis at T0 were evaluated using the reference values of PSEP in healthy term and preterm neonates previously reported [15]. The overall AUC of PSEP (Figure 2A and Table 3) was 0.862 (95% CI: 0.828–0.896), with a maximum Youden Index (best cut-off point) at 987.5 pg/mL, corresponding to a sensitivity of 72% and a specificity of 87%.

The negative predictive value at the best cut-off point was 0.93. After stratification for clinical severity (infection, sepsis, septic shock), the AUC progressively increased with worsening clinical conditions (Figure 2B, Table 3, and Supplementary Table S1). The maximum Youden Index was 687.5 pg/mL for infection (81% sensitivity, 62% specificity), 1013 pg/mL for sepsis (84% sensitivity, 88% specificity), and 971.5 pg/mL for septic shock (92% sensitivity, 86% specificity).

Then, PSEP, CRP and PCT were benchmarked for their ability to discriminate neonates with severe clinical courses (sepsis and septic shock) from those with a milder course (infection) after the onset of symptoms (Figure 2C–E). ROC curves showed an AUC of PSEP for sepsis of 0.73 (95% CI: 0.647–0.817) and for septic shock of 0.79 (95% CI: 0.683–0.9) when neonates with infection were used as reference. Conversely, CRP and PCT had limited value to discriminate neonates with the most severe clinical courses already at T0, with a limited utility of PCT for the early discrimination of patients subsequently developing septic shock (AUC septic shock vs. infection 0.65, 95% CI: 0.504–0.795). Overall, PSEP had a better diagnostic accuracy for sepsis and septic shock compared to CRP and PCT.

### 2.3. Presepsin Correlation with Positive Blood Culture

**Figure 3:**
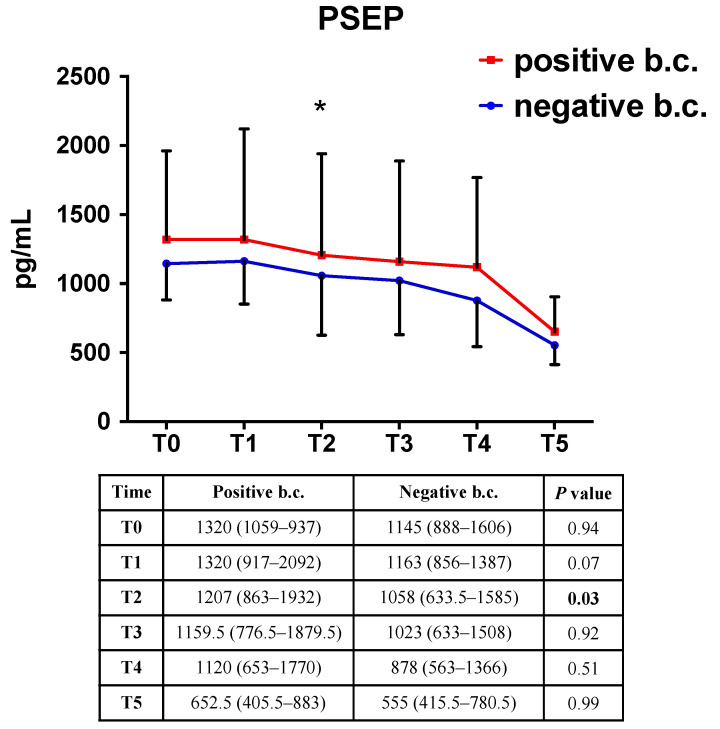
Serial values (median, (IQR)) of PSEP in neonates with positive and negative blood culture. Repeated measures ANOVA with Tukey’s correction for multiple comparisons. *, *p* < 0.05. b.c.: blood culture.

At T0, neonates with a positive blood culture had a median PSEP concentration slightly, but not significantly, higher than neonates with negative blood culture (1320 vs. 1145 pg/mL, *p* = 0.94) (Figure 3). Similarly, almost overlapping values of CRP and PCT between the two groups were detected at T0 (Supplementary Figure S3). PSEP median values became significantly higher in neonates with positive blood culture, compared to those with negative blood culture, 24 h after the onset of symptoms (T2, 1207 vs. 1058, *p* = 0.03). Conversely, neither CRP nor PCT could effectively discern the two groups over time (Supplementary Figure S4).

## 3. Discussion

In the present study, we aimed to investigate whether PSEP could support the early identification, among neonates with symptoms suspicious for sepsis, of: (1) those who subsequently develop the most severe clinical conditions; and (2) those with a positive blood culture. An early, reliable attribution of neonates to one or both these categories would improve the tailoring of early clinical approaches, which could allow the sparing of unnecessary (or unnecessarily long) antibiotic therapies, notoriously associated with several adverse consequences, especially for VLBW infants [17,18].

Here, we found that PSEP at the onset of symptoms was significantly associated with clinical severity over the following 48 h. The stepwise increase in PSEP levels according to clinical severity resembled the results of several published studies on adult patients [19,20,21], with variations in part due to different definitions of sepsis and septic shock [22]. Studies on neonates also show inconsistency and, according to four recent meta-analyses [11,12,13,14], major limitations of most studies on PSEP for the diagnosis of neonatal sepsis are the absence of stratification for clinical severity and the case–control design, in which neonates with confirmed neonatal sepsis are only compared to healthy controls. Indeed, net of two studies [23,24] marginally addressing the issue of neonatal septic shock, this is the first detailed analysis of PSEP diagnostic accuracy according to rigorous and clinically relevant definitions of neonatal sepsis and septic shock [6]. 

When we compared the whole group of neonates with symptoms suspicious for sepsis with our historical controls [15], we obtained an overall AUC at T0 of 0.862, which is an intermediate value between the highest areas for neonates with shock (0.94) or sepsis (0.90) and the lowest for neonates with milder course (0.78). The prompt ability of PSEP to discriminate neonates with a severe course from those with mild symptoms over the next two days was also confirmed when we built ROC curves using neonates with mild clinical course as the reference group. Here, we obtained lower AUCs, equal to 0.73 for sepsis and 0.79 for septic shock. These values were greatly superior to the corresponding diagnostic performance of CRP and PCT, which could not effectively distinguish the three groups of enrolled neonates. Interestingly, these lower AUC values are comparable to those reported in a small, similarly designed trial on neonates [25]. We report a maximum Youden index for the diagnosis of sepsis at 1013 pg/mL, and for the diagnosis of septic shock at 971.5 pg/mL. The slightly higher value for sepsis compared to septic shock may be due to the limited number of neonates enrolled in the shock group, and to the fact that the two ROC curves frequently overlap. These thresholds were higher than those reported by others (between 650 and 850 pg/mL) [11,12], but supported by the use of control values from the largest cohort of healthy term and preterm neonates used to establish the reference ranges [15], which reported average concentrations of PSEP in healthy term and preterm neonates of 604 and 620 pg/mL, respectively. Thus, the previously suggested threshold of 650 pg/mL to rule-in neonatal sepsis seems too close to these reference values in order to be of some clinical utility. 

Despite its good performance in discriminating severe clinical courses, we could not demonstrate a significant difference in PSEP values at the onset of symptoms between neonates with a positive or negative blood culture. Published studies on neonates showed conflicting results. Some authors reported significantly higher levels of PSEP in cases of culture-proven sepsis [26,27,28], while others failed to find a significant difference between culture-proven and clinical sepsis [29]. In our cohort, neonates with positive blood culture showed significantly higher PSEP levels only after 24 h from the onset of symptoms. Furthermore, the slight absolute difference between neonates with positive and negative blood culture may be of questionable clinical utility in a real-life scenario. Therefore, we conclude that PSEP cannot be used alone to rule out culture-positive sepsis nor to decide not to start empiric antibiotic therapy. However, serial evaluations of PSEP, if our results were to be confirmed by larger cohorts, might support the early interruption of unnecessary antibiotic therapy. Finally, in our population, only 4% of neonates died from sepsis; thus, we could not perform a meaningful analysis on prognostic value for death. It may be noteworthy, nonetheless, to evaluate the role of PSEP as a prognostic marker of adverse long-term sepsis-related outcomes, but further analyses and larger cohorts are required. 

Significant strengths of our study are the rigorous definition and stratification, according to clinical severity, of neonatal sepsis, as well as the comparison of symptomatic neonates with a large, previously validated cohort of healthy term and preterm neonates. However, even though our cohort of neonates with suspected sepsis is among the largest reported in the literature, it is still limited compared to most cohorts of adult patients. Therefore, larger, possibly multi-center collaborative trials are necessary to further deepen our understanding of the role of PSEP in the context of neonatal sepsis.

## 4. Materials and Methods

### 4.1. Study Design and Inclusion Criteria

This was a prospective, double-center diagnostic trial conducted at the NICUs of Fondazione IRCCS Ca’ Granda Ospedale Maggiore Policlinico, University of Milan, Italy, and of Careggi University Hospital of Florence, Italy, over a 24 month period. The study was approved by the Institutional Review Boards of the two participating institutions.

Neonates of any gestational age (GA) with a first episode of suspected sepsis, either early-onset (EOS) or late-onset sepsis (LOS), were consecutively enrolled. Those with severe congenital anomalies were excluded. After enrollment, neonates received standard therapy for suspected neonatal sepsis, including broad spectrum antibiotics, fluid resuscitation, and vasoactive agents as required. Whole blood concentrations of PSEP were assessed at the onset of clinical signs of sepsis (T0), every 12 h for the following 48 h (T1, T2, T3, T4), and at the end of antibiotic therapy (T5). CRP and PCT were measured at T0, T2, T4 and T5. All data were recorded in an electronic database. 

### 4.2. Definitions

Suspected neonatal sepsis was defined according to CDC criteria [30], in the presence of at least one clinical symptom plus the need for antibiotic therapy upon physician’s evaluation. Enrolled neonates were then classified into 3 groups according to Wynn et al.’s definitions [6]: group 1, infection (suspected infection not meeting the criteria for sepsis); group 2, sepsis (neonatal systemic inflammatory response syndrome, “SIRS”, plus suspected or proven infection); group 3, septic shock (sepsis plus cardiovascular organ dysfunction). The definitions of SIRS, cardiovascular dysfunction and organ dysfunction provided by Wynn et al. were applied [6]. Proven sepsis was defined by the occurrence of at least one blood culture positive for bacteria, including coagulase-negative Staphylococci, in addition to clinical signs suggestive of infection. Sepsis-related mortality was defined as death occurring within 7 days from the onset of clinical symptoms and not attributable to other major coexisting causes [31].

### 4.3. Collection of Samples and Measurement of Biomarkers

PSEP was measured on 100 microliters of blood collected in ethylenediaminetetraacetic acid (EDTA) tubes and processed within 4 h from the withdrawal. A chemiluminescence enzyme immunoassay was used (PATHFASTTM System, LSI Medience Corporation, Tokyo, Japan). PSEP concentration was corrected for the hematocrit value. CRP was quantified using a standardized immune-turbidimetric assay (Cobas^®^, Roche, Monza, Italy), PCT by an immune-chemical assay (Cobas^®^, Roche, Monza, Italy). Blood culture was obtained simultaneously to PSEP measurement: at least 1 mL of blood was seeded in aerobic BD BactecTM Peds PlusTM medium bottles (Becton, Dickinson and Company, Franklin Lakes, NJ, USA). When possible, 2 cultures were obtained from different peripheral sites.

### 4.4. Statistical Analysis

Continuous variables are presented as the mean (standard deviation, SD) or median (interquartile range, IQR), according to their distribution. Categorical variables are presented as absolute frequencies (percentages). Differences between groups in continuous variables were assessed by one-way ANOVA with Dunn’s multiple comparisons test or by Kruskal–Wallis test, as appropriate. Categorical variables were compared with a chi-squared test. To compare the kinetics of PSEP, CRP and PCT over time and between groups, repeated-measures (RM) ANOVA with Tukey’s test for multiple comparison was applied. Diagnostic accuracy of PSEP was evaluated with the area under the receiver-operating characteristic (ROC) curve (AUC). Sample size was not determined a priori.

SAS version 9.4, SAS Institute Inc., Cary, NC, USA and GraphPad Prism version 8.0 (GraphPad Software Inc., San Diego, CA, USA) software were used. A two-sided *p*-value of less than 0.05 was considered statistically significant.

## 5. Conclusions

In conclusion, our data showed that PSEP is an accurate biomarker for the timely identification of septic neonates at higher risk for a rapid derangement of clinical conditions, favoring a tailored medical and therapeutic approach. Conversely, in our limited cohort, PSEP alone was not able to distinguish neonates with a subsequent positive from those with a negative blood culture at the onset of clinical symptoms, however, if confirmed in larger populations, it may support an “early discontinuation” strategy of empiric antibiotic therapy. This study may lay the foundation for interventional trials on large cohorts of neonates to establish the role of PSEP, alone or in combination with other biomarkers, in driving medical decision-making. 

## Figures and Tables

**Table 1:** Baseline characteristics and T0 data (where specified) of neonates enrolled. GA: gestational age. BW: birth weight. SGA: small for gestational age. ETT: endotracheal tube. CVC: central venous catheter. *p*-values were calculated by one-way ANOVA (continuous variables) and chi-squared test (categorical variables).

	Group 1 (*n* = 58)	Group 2 (*n* = 77)	Group 3 (*n* = 24)	*p*-Value
	Infection	Sepsis	Septic Shock	
GA, mean (SD), weeks	33.8 (5.8)	31 (5.2)	30 (4.5)	0.002
BW, mean (SD), grams	2205 (1224.8)	1612.2 (1065.5)	1425.8 (921.7)	0.002
Male, *n* (%)	33 (56.9)	52 (67.5)	12 (51)	0.22
SGA, *n* (%)	10 (17.2)	16 (20.8)	4 (16.7)	0.84
Clinical chorioamnionitis, *n* (%)	6 (10.4)	9 (11.7)	4 (16.7)	0.73
Apgar score at 5 min, median, (IQR)	9 (8–10)	8 (8–9)	8 (7–9)	0.003
Age at T0, median (IQR)	2 (1–22.3)	23 (12–37.5)	10.5 (1–29.8)	<0.001
ETT at T0, *n* (%)	5 (8.9)	28 (38.9)	14 (58.3)	<0.001
CVC at T0, *n* (%)	19 (33.9)	51 (70.8)	19 (79.2)	<0.001
No. of clinical signs at T0, median (IQR)	2 (1–3)	4 (3–5)	6 (5–8)	<0.001
Fever at T0, *n* (%)	1 (1.7)	35 (45.5)	12 (50)	<0.001
Oligoanuria at T0, *n* (%)	0	2 (2.6)	5 (20.8)	<0.001
White blood cells at T0, median (IQR), cell * 10^9^/L	13,415 (9150–18,620)	12,230 (7240–20,180)	5120 (2800–14,080)	0.072
Platelets at T0, mean (SD), *10^9^/L	266 (134)	248 (142)	197 (152)	0.125
Lactate at T0, mean (SD), mmol/L	2.8 (2)	2.1 (1.8)	3.5 (2.3)	0.004
Inotropic drugs at T0, *n* (%)	2 (3.4)	6 (7.8)	20 (83.3)	<0.001
Hydrocortisone at T0, *n* (%)	0	2 (2.6)	13 (54.2)	<0.001
Positive blood culture at T0, *n* (%)	15/58 (25.8)	46/77 (59.7)	16/24 (66.7)	<0.001
Gram-negative bacteria, *n* (%)	1 (1.72)	16 (20.8)	8 (33.3)	<0.001
Days of antibiotic therapy, mean (SD)	9 (4.4)	12 (5.6)	13 (6.9)	0.001
Sepsis-related mortality, *n* (%)	0	4/77 (5.2)	3/24 (12.5)	0.042
Presepsin at T0, median (IQR), pg/mL	977.5 (709–1239)	1361 (1082–2065)	1557.5 (1149.5–2386)	<0.001
CRP at T0, median (IQR), mg/dL	3.8 (1.5–6.3)	3.8 (1–6.7)	3.4 (0.7–7.9)	0.49
PCT at T0, median (IQR), ng/mL	3 (0.8–17)	2.4 (0.8–14.3)	19.8 (1.8–31.4)	0.14

**Table 2:** Serial values (median, (IQR)) of PSEP, CRP and PCT in the three groups of neonates enrolled. “Adjusted *p*-value 1” refers to multivariable linear regression adjusted for GA and BW of Sepsis vs. Infection. “Adjusted *p*-value 2” refers to multivariable linear regression adjusted for GA and BW of Septic Shock vs. Infection.

Time	Infection	Sepsis	Septic Shock	Adjusted *p*-Value 1	Adjusted *p*-Value 2
	**PSEP (pg/mL)**				
T0	977.7, (709–1239)	1361, (1082–2065)	1557.5, (1149.5–2386)	<0.001	<0.001
T1	957, (782–1233)	1311, (961.5–1851)	1645, (1182–2366)	0.001	<0.001
T2	875, (709–1227)	1279, (759–1801)	1789, (1113–2618)	0.004	<0.001
T3	844.5, (633.0–1083)	1159.5, (703–1874.5)	1713.0, (1065–3087)	0.002	<0.001
T4	772.5, (458–1141.0)	1072.5, (799.0–1741)	1740.0, (782–2547)	<0.001	<0.001
T5	528.0, (413–677)	638.5, (372–929)	681, (603–1468)	0.91	0.13
	**CRP (mg/dL)**				
T0	3.8, (1.5–6.3)	3.8, (1–6.7)	3.4, (0.7–7.9)	0.139	0.224
T1					
T2	2.6, (1.4–5.5)	5.9, (2.2–11.3)	8.2, (4.2–14.9)	0.001	<0.001
T3					
T4	1.3, (0.7–3.5)	3.7, (1.1–8.7)	7.5, (1.2–11.1)	0.001	0.007
T5	0.5, (0.3–0.7)	0.5, (0.2–0.7)	0.5, (0.2–0.7)	0.266	0.537
	**PCT (ng/mL)**				
T0	3, (0.8–17)	2.4, (0.8–14.3)	19.8, (1.8–31.4)	0.755	0.031
T1					
T2	2.2, (0.6–19.4)	4.3, (1.3–23)	18.5, (4.5–46.5)	0.442	0.026
T3					
T4	0.8, (0.5–4.5)	3.1, (0.9–10.8)	7.7, (4–48.3)	0.31	0.001
T5	0.2, (0–0.3)	0.2, (0.1–0.5)	0.3, (0.1–0.3)	0.454	0.874

**Table 3:** Diagnostic accuracy of PSEP at T0 using healthy neonates as a reference and then the neonates of the “infection” group. PPV: positive predictive value. NPV: negative predictive value. POS LR: positive likelihood ratio. NEG LR: negative likelihood ratio. Confidence intervals are in brackets.

	Max. Youden Index (pg/mL)	Sensitivity	Specificity	PPV	NPV	POS LR	NEG LR
**Reference Group: Healthy Neonates**							
**OVERALL**	**987.5**	**0.72**	**0.87**	**0.57**	**0.93**	**5.65 (4.54–7.02)**	**0.32 (0.25–0.41)**
**Infection**	687.5	0.81	0.62	0.15	0.98	2.16 (1.84–2.53)	0.30 (0.18 to 0.52)
**Sepsis**	1013	0.84	0.88	0.45	0.98	7.16 (5.71–8.97)	0.17 (0.10–0.30)
**Septic shock**	971.5	0.92	0.86	0.18	1.00	6.42 (5.16–8.00)	0.09 (0.03–0.37)
**Reference Group: “Infection” Group**							
**Sepsis**	1006	0.84	0.55	0.71	0.73	1.88 (1.39–2.55)	0.28 (0.16–0.50)
**Septic shock**	1139	0.83	0.64	0.49	0.90	2.3 (1.57–3.38)	0.26 (0.10–0.65)

## Data Availability

The anonymized datasets used during the current study are available from the corresponding author on reasonable request.

## Supplementary Materials

The following are available online at https://www.mdpi.com/article/10.3390/antibiotics10050580/s1, Figure S1: CRP and PCT values at T0 in the three groups of enrolled neonates, Figure S2: Correlations between presepsin values at T0 and corresponding values of CRP or PCT in the three groups of enrolled neonates, Figure S3: CRP and PCT values at T0 in neonates with negative and positive blood culture, Figure S4: CRP and PCT kinetics over time in neonates with negative and positive blood culture, Table S1: Diagnostic performance of PSEP for the diagnosis of infection, sepsis and septic shock at different cut-off values overall, after stratification for clinical severity and considering the “infection” group as reference.
