# Supplementary material for: Presepsin (Soluble CD14 Subtype) as an Early Marker of Neonatal Sepsis and Septic Shock: A Prospective Diagnostic Trial
Source: Antibiotics (Basel). 2021 May 14;10(5):580. doi: 10.3390/antibiotics10050580 (PMC8156848; doi:10.3390/antibiotics10050580)
Supplement: Supplementary file 1 [file antibiotics-10-00580-s001.zip › antibiotics-1178178-supplementary.pdf]

Suppementary material

Presepsin (soluble CD14 subtype) as an early marker of clinical severity in neonatal sepsis and septic shock: a prospective diagnostic trial

Authors: Pietrasanta C, Ronchi A, et al.

Figure S1. CRP and PCT values at T0 in the three groups of enrolled neonates.

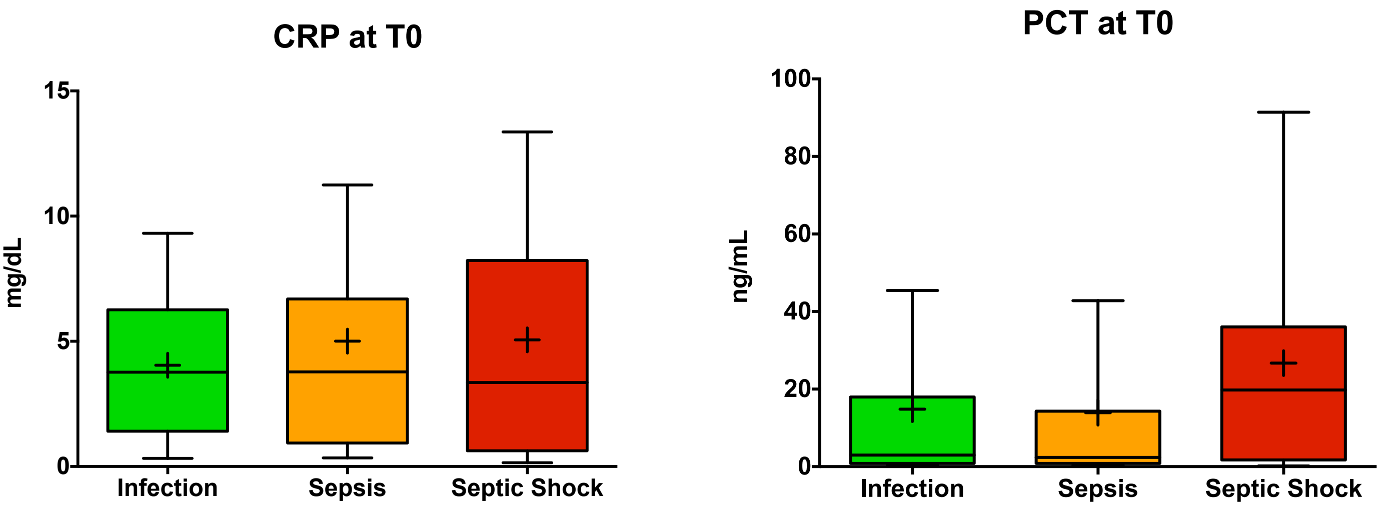

In each graph, boxes indicate interquartile range, whiskers indicate 10° and 90° percentile, crosses indicate means. **=p <0.01 after ANOVA with Dunn's multiple comparisons test.

Figure S2. correlations between presepsin values at T0 and corresponding values of CRP or PCT in the three groups of enrolled neonates.

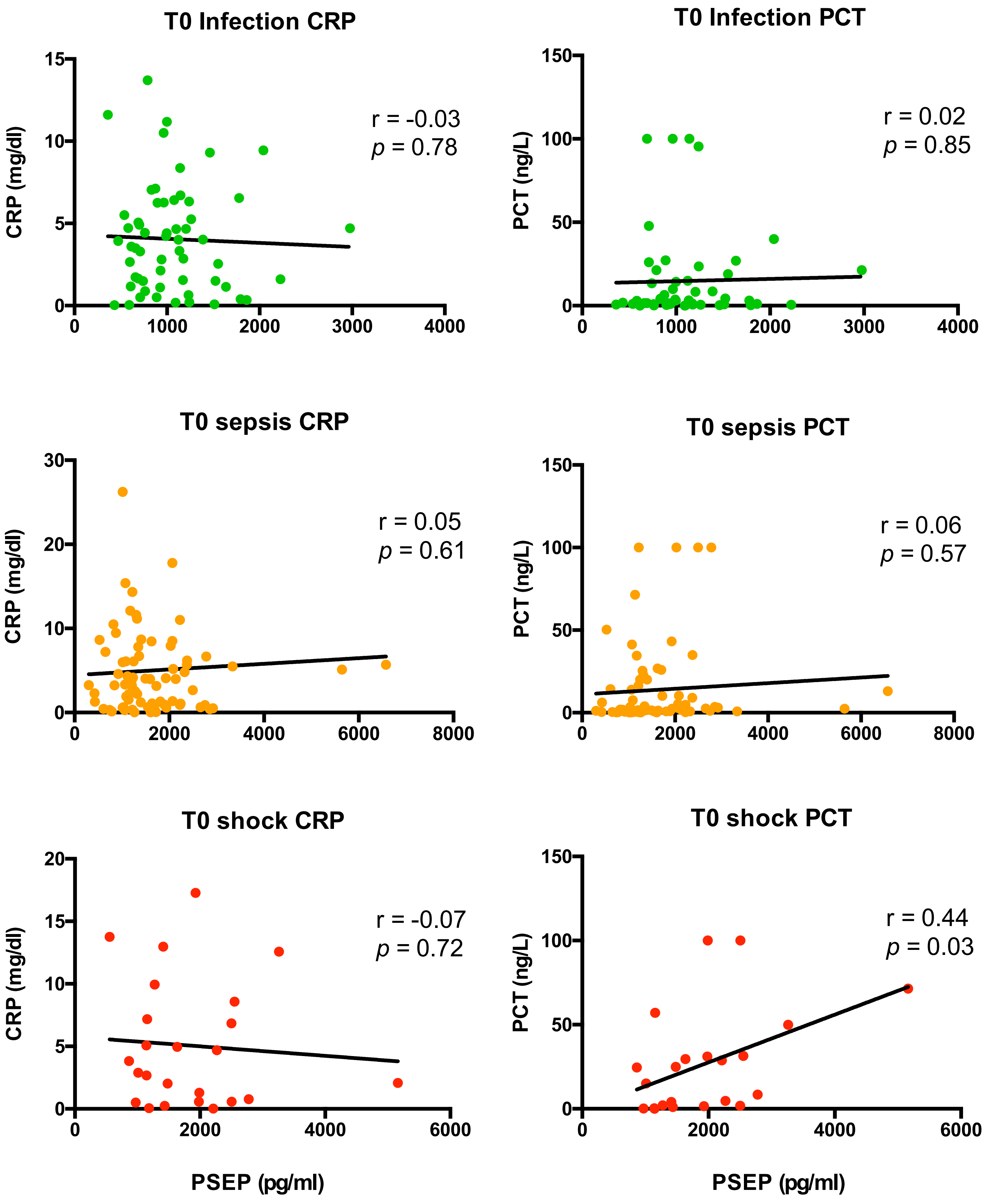

Figure S3. CRP and PCT values at T0 in neonates with negative and positive blood culture.

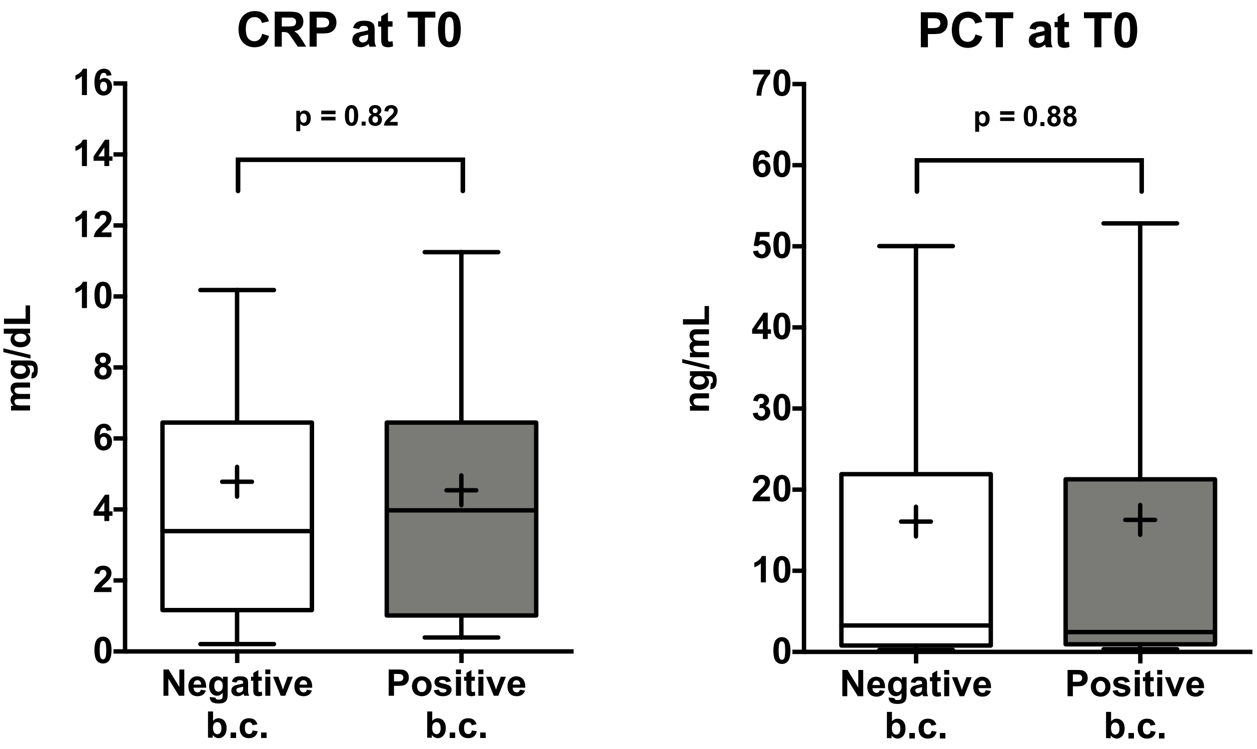

In each graph, boxes indicate interquartile range, whiskers indicate 10° and 90° percentile, crosses indicate means. *P* values after Mann-Whitney U test. B.c.: blood culture

Figure S4. CRP and PCT kinetics over time in neonates with negative and positive blood culture.

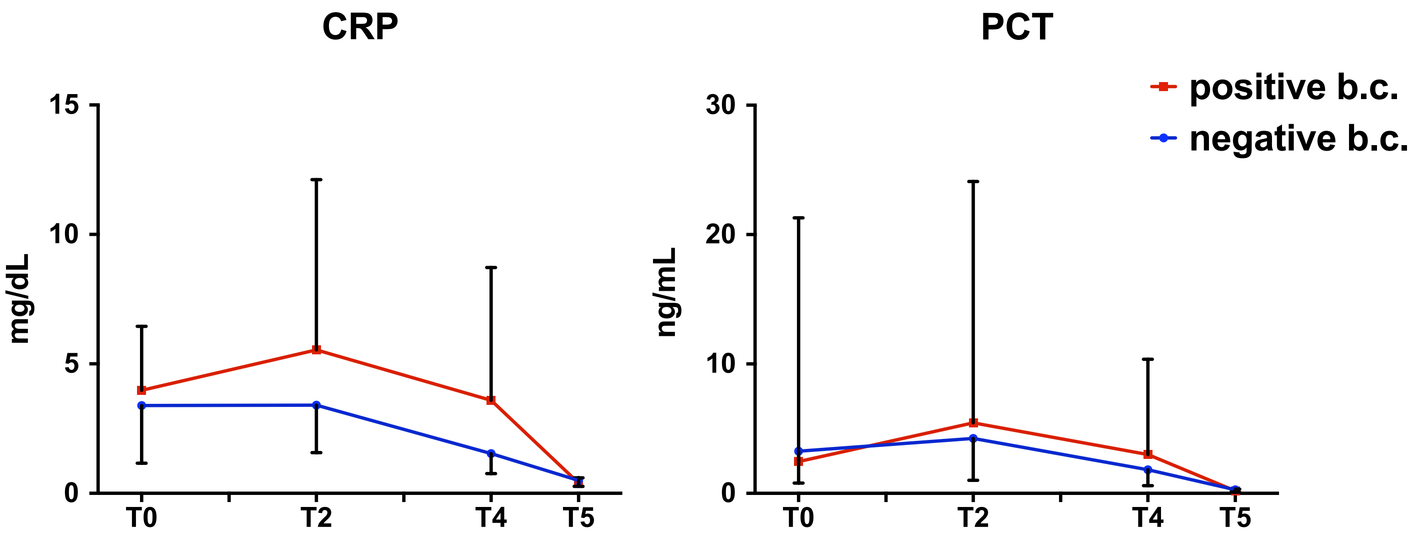

For each biomarker, median with interquartile range is depicted. B.C.: blood colture

Table S1. Diagnostic performance of PSEP for the diagnosis of infection, sepsis and septic shock at different cut-off values overall, after stratification for clinical severity and considering the "infection" group as reference. PPV: positive predictive value. NPV: negative predictive value. POS LR: positive likelihood ratio. NEG LR: negative likelihood ratio

	Cut-off (pg/mL)	Sensitivity	Specificity	PPV	NPV	POS LR	NEG LR
**Reference group: Healthy neonates**							
**Infection *vs.* Controls**	**>650**	0.84	0.57	0.14	0.98	1.95 (1.69 -2 .24)	0.27 (0.15 - 0.50)
	**>800**	0.66	0.74	0.17	0.96	2.49 (1.99 - 3.11)	0.47 (0.33 - 0.67)
	**>950**	0.52	0.85	0.22	0.95	3.50 (2.60 - 4.71)	0.55 (0.42 - 0.73)
	**>1100**	0.40	0.91	0.26	0.95	4.26 (2.87 - 6.31)	0.67 (0.54 - 0.82)
	**>1250**	0.22	0.95	0.29	0.94	4.81 (2.68 - 8.65)	0.81 (0.71 - 0.94)
	**>1500**	0.17	0.99	0.63	0.93	19.74 (7.44-52.39)	0.83 (0.74 - 0.94)
**Sepsis *vs.* Controls**	**>650**	0.92	0.57	0.19	0.98	2.13 (1.91 - 2.37)	0.14 (0.06 - 0.30)
	**>800**	0.90	0.74	0.28	0.98	3.40 (2.94 - 3.94)	0.14 (0.07 - 0.27)
	**>950**	0.84	0.85	0.38	0.98	5.52 (4.52 - 6.75)	0.18 (0.11 - 0.31)
	**>1100**	0.73	0.91	0.47	0.97	7.81 (5.96-10.23)	0.30 (0.21 - 0.43)
	**>1250**	0.58	0.95	0.58	0.95	12.5 (8.52-18.48)	0.44 (0.33 - 0.57)
	**>1500**	0.45	0.99	0.85	0.94	52.0 (22.62-119.76)	0.55 (0.45 - 0.67)
**Septic shock *vs.* Controls**	**>650**	0.96	0.57	0.07	1.00	2.21 (1.96 - 2.49)	0.07 (0.01 – 0.50)
	**>800**	0.96	0.74	0.11	1.00	3.64 (3.13 - 4.23)	0.06 (0.01 - 0.39)
	**>950**	0.92	0.85	0.17	1.00	6.0 (4.84 - 7.42)	0.10 (0.03 - 0.37)
	**>1100**	0.83	0.91	0.24	0.99	8.95 (6.67-12.00)	0.18 (0.08 - 0.45)
	**>1250**	0.67	0.95	0.33	0.99	14.3 (9.21-22.25)	0.35 (0.20 - 0.62)
	**>1500**	0.50	0.99	0.67	0.98	57.2 (23.48 -139.62)	0.50 0.34 - 0.75
**Reference group: "Infection" group**							
**Sepsis *vs.* Infection**	**>650**	0.92	0.16	0.59	0.60	1.09 (0.96 - 1.24)	0.5 (0.19 - 1.33)
	**>800**	0.90	0.34	0.64	0.71	1.37 (1.12 - 1.67)	0.30 (0.14 - 0.64)
	**>950**	0.84	0.47	0.68	0.69	1.58 (1.22 - 2.05)	0.33 (0.19 - 0.60)
	**>1100**	0.73	0.60	0.71	0.63	1.83 (1.30 - 2.59)	0.46 (0.30 - 0.69)
	**>1250**	0.58	0.78	0.78	0.58	2.7 (1.56 - 4.36)	0.54 (0.40 - 0.72)
	**>1500**	0.45	0.83	0.78	0.53	2.63	0.66
**Septic shock *vs.* Infection**	**>650**	0.96	0.16	0.32	0.90	1.13 (0.99 - 1.30)	0.27 (0.04 - 2.01)
	**>800**	0.96	0.34	0.38	0.95	1.46 (1.19 - 1.79)	0.13 (0.02 - 0.85)
	**>950**	0.92	0.47	0.42	0.93	1.72 (1.31 - 2.24)	0.18 (0.05 - 0.69)
	**>1100**	0.83	0.60	0.47	0.90	2.1 (1.46 - 3.03)	0.27 (0.11 - 0.69)
	**>1250**	0.67	0.78	0.55	0.85	2.97 (1.71 - 5.19)	0.43 (0.24 - 0.77)
	**>1500**	0.50	0.83	0.55	0.80	2.9 (1.45 - 5.79)	0.6 (0.40 - 0.92)
